# On-Device Deep Personalization for Robust Activity Data Collection †

**DOI:** 10.3390/s21010041

**Published:** 2020-12-23

**Authors:** Nattaya Mairittha, Tittaya Mairittha, Sozo Inoue

**Affiliations:** Graduate School of Engineering, Kyushu Institute of Technology, 1-1 Sensui-cho, Tobata-ku, Kitakyushu-shi, Fukuoka 804-8550, Japan; callmefons@gmail.com (T.M.); sozo@sozolab.jp (S.I.)

**Keywords:** activity recognition, data collection, on-device personalization, deep learning, fine-tuning, smartphone sensors, user feedback

## Abstract

One of the biggest challenges of activity data collection is the need to rely on users and keep them engaged to continually provide labels. Recent breakthroughs in mobile platforms have proven effective in bringing deep neural networks powered intelligence into mobile devices. This study proposes a novel on-device personalization for data labeling for an activity recognition system using mobile sensing. The key idea behind this system is that estimated activities personalized for a specific individual user can be used as feedback to motivate user contribution and improve data labeling quality. First, we exploited fine-tuning using a Deep Recurrent Neural Network to address the lack of sufficient training data and minimize the need for training deep learning on mobile devices from scratch. Second, we utilized a model pruning technique to reduce the computation cost of on-device personalization without affecting the accuracy. Finally, we built a robust activity data labeling system by integrating the two techniques outlined above, allowing the mobile application to create a personalized experience for the user. To demonstrate the proposed model’s capability and feasibility, we developed and deployed the proposed system to realistic settings. For our experimental setup, we gathered more than 16,800 activity windows from 12 activity classes using smartphone sensors. We empirically evaluated the proposed quality by comparing it with a baseline using machine learning. Our results indicate that the proposed system effectively improved activity accuracy recognition for individual users and reduced cost and latency for inference for mobile devices. Based on our findings, we highlight critical and promising future research directions regarding the design of efficient activity data collection with on-device personalization.

## 1. Introduction

Mobile activity recognition is mostly implemented using supervised learning algorithms. The training of these supervised algorithms challenges labeled data or “ground truth.” Incorrect or unfinished labeling may result in classification failures that lead to inaccurate systems; hence, achieving high-quality labels is crucial. Data labeling using smartphone sensors can be done in several ways, depending on the nature of data being labeled. Both ways impose challenges [1,2]. In this study, we challenge the online and self-labeling scenarios using inertial sensors, such as accelerometers. Data labeling is labeled when the individual is performing the activity of concern. Human labelers must start and stop the data capture process manually to label describing the on-going activity that needs to be assessed to avoid inaccurate timestamps, which requires high effort. Although participants show initial enthusiasm, they may lose interest and drop out over time. This situation leads to low-quality data collection and biased data. Indeed, it is hard to overcome the lack of motivation and sustained engagement without any artifice [3,4,5,6]. Thus, our motivation is to create a strategy to keep the participants engaged with the labeling task to obtain high-quality labels.

This challenge is well-motivated in user feedback studies. [3,7,8]. Prior work [8] utilized inference results as feedback to improve the quality and quantity of participant contributions. However, the datasets’ models from different users lose accuracy when applied to a new user due to the diversity of users’ behavior. This limitation can be addressed either by training a personal model on a cloud or a device. On-cloud training has the disadvantage of high computational cost and inability to scale when training the per-user model for millions of users. Contrarily, on-device training might give model training’s inefficiency because of resource-constrained devices and insufficient user data on-device.

This study bridges this gap by introducing the proposed system, allowing activity recognition applications for smartphone sensor systems to achieve highly accurate training datasets based on three features. First, we employ *Fine-tuning deep neural networks* [9]: the technique widely used in transfer learning in the context of deep learning to overcome the lack of sufficient training data. We implement fine-tuning instead of full-training, called on-device personalization, helping models stay relevant to user behavior. Second, we propose *Magnitude-based weight pruning* [10]: an optimization technique to minimize the complexity of optimizing deep learning inference for on-device personalization. Finally, we integrate the abovementioned two features to build an efficient on-device personalization system. We utilize the inference results obtained from the on-device model as feedback to motivate user engagement and improve data labeling quality.

**Figure 1:**
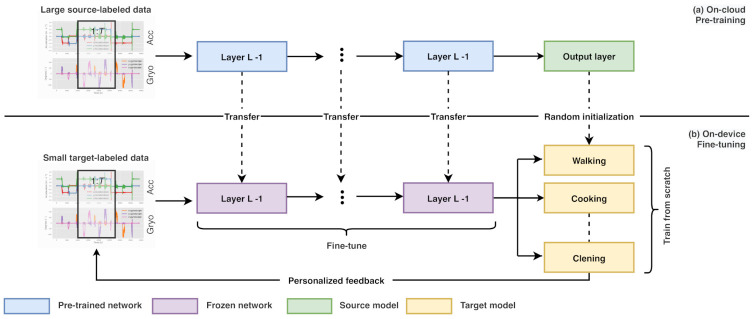
High-level overview of the proposed system. We train RNNs for activity recognition on an extensive labeled dataset Step (**a**). The learned features are transferred to the below activity recognition model on a device Step (**b**) to personalize individual devices’ prediction with a small labeled dataset. Next, the predicted activities are continuously returned as feedback for data labeling.

In short, the proposed system focuses on the accuracy of human contributions in achieving high-quality and consistent ground-truth labeling and, particularly, on the impact of the “on-device personalization system” and feedback under different conditions (See Table 1). To be entirely sure, the experimental setup was a within-subject design; the same person tests all the conditions where each participant receives both with- and without feedback. An overview of the proposed system is shown in Figure 1. The contributions of this work to the field are the following:

1. We introduce a system design of integrating on-device personalization and activity recognition, which allows activity recognition applications for smartphone sensor systems to achieve highly accurate training datasets. We developed the proposed system based on three essential features: on-device fine-tuning, model optimization, and personalized feedback.
2. We deployed the proposed system to a realistic scenario demonstrating its capability and feasibility. We gathered more than 16,800 activity windows, each labeled with their corresponding activity class from 12 activity classes using smartphone sensors. We reviewed, analyzed, and used the obtained data for evaluations.
3. We empirically evaluated the proposed system’s quality by comparing the proposed condition with the baseline condition (see Table 1) using machine learning. The results indicate that the proposed system can achieve accurate and consistent labeling in activity datasets.

We discuss the results, challenges, limitations, and implications of this research on the design of efficient activity data collection methods with on-device personalization.

## 2. Related Work

This section discusses existing literature studies that relate to our work in this study. We first review the background and challenges of label collection for activity recognition. We then introduce two key ideas that drive our research: (1) on-device deep learning; (2) decentralized machine learning.

- **Challenges of data labeling for activity recognition:** Presently, the principal activity recognition models produced require manually labeled data by a human in a way that allows them to learn how to build correct decisions. Label collection for activity recognition with smartphone sensors has various challenges concerning four different criteria. First, either data labeling was done by online [11,12] or by offline [13]; they must achieve highly accurate timestamps and overcome extended memorization. Second, either data labeling was done by self-labeling or by an observer; they impose several challenges such as missing labels, inaccurate timestamps, and high cost [12,14,15]. Third, either data labeling was done in a laboratory scenario or a realistic scenario; they present specific challenges. For example, the models produced in laboratory settings lose accuracy when applied in real circumstances due to the variety of users’ behavior. In comparison, the models produced in practical environments tend to be more generic but complicated [15,16,17,18,19]. Finally, there are many challenges to all data labeling mechanisms undertaken. For instance, the use of domain experts’ to manually label data typically results in more truthful labeling, but it can be high-priced and time-consuming. In contrast, the use of fully automated labeling mechanisms can reduce time but may not be as precise as those delivered by a domain expert [20,21,22]. This study challenges the online and self-labeling scenarios in a realistic setting.
- **On-device deep learning**: Deep learning with ubiquitous technologies is increasingly considered by researchers, particularly for mobile devices [23,24]. With the powerful mobile devices’ hardware, it is possible to exploit deep learning to solve a problem using a mobile device and its sensors to collect data without cloud support. The cloud-based approach can reach almost infinite resources, but there is a long delay between data collection and model updates. Contrarily, the mobile-based approach can answer the drawbacks of the cloud model by running some or all model training to the device itself. Consequently, the use of deep learning on mobile devices has been researched in many works [25,26,27,28,29]. The use of knowledge transfer for on-device deep learning has been the subject of study of some works [30]. However, there are some critical drawbacks concerning deep learning methods on resource-constrained devices [31]. Some present works have been proposed to build deep learning that is effective on resource-constrained devices, such as model compression [26,29,32,33,34] and customized hardware design assistance [35,36,37]. Some of these works are utilized in our work (e.g., layer compression), but they mostly target only the inference phase of deep learning algorithms. Contrarily, we introduce a technique to minimize the complexity of optimizing on-device deep learning inference.
- **Decentralized machine learning:** With the advent of connected devices with computation and storage capabilities, running machine learning workflows on-device is possible. Unlike standard machine learning solutions, decentralized machine learning [38,39,40,41] distributes the learning phase over distributed networks of devices. For instance, Konečnỳ et al. [39] explored federated learning in which users do not send the data they generate to a data center at all, but rather provide part of their computational power to solve optimization problems. Our study exploits fine-tuning training where the locally trained models or parameter updates will not be uploaded to the cloud as we already trained and generalized the global model. This solution improves upon the traditional approaches by working better in bandwidth and power-constrained environments and provides a straightforward and effective mechanism for personalization at scale.

## 3. Preliminaries

This section provides a brief overview of multiple learning paradigms, including mobile activity recognition with deep learning, transfer learning and fine-tuning, and, importantly, on-device personalization.

### 3.1. Mobile Activity Recognition with Deep Learning

This study relies on state-of-the-art mobile activity recognition using supervised learning, the input *x* is sensor data (regularly represented as a set of sensor input values around time *t*). We typically describe an example as a vector $x\in R^{n}$, where each $x_{i}$ of the vectors is a different feature. The output *y* is a numeric value classifying the activity class *k* in the given sensor data. The learning algorithm must produce a function $f:R^{n}\to \left\{1,\cdots ,k\right\}$. When y=f(x), the model assigns an input defined by vector *x* to a category *k* defined by numeric value *y*, where *f* can output a probability distribution over classes. Recent activity recognition is well-developed with deep learning [42] to overcome traditional algorithms’ failure on such recognition tasks. The deep learning strategy is to learn ϕ, where ϕ can be used as a provided set of features characterizing *x* or a new representation for *x*. In this strategy, we have a model $y=f\left(x;\theta ,\omega \right)=\phi {\left(x;\theta \right)}^{T}\omega$. We have parameters θ that we apply to learn ϕ from a broad class of functions, and parameters ω that map from ϕω to the desired output. This is an instance of a common deep learning, where ϕ defining a hidden layer. We parametrize the representation as ϕ(x;θ) and utilize the optimization algorithm to find the value of the parameters θ that result in the most useful function approximation.

The use of Convolutional neural networks (CNNs) and Recurrent neural networks (RNNs) have been the subject of study of many activity recognition applications [43]. Both kinds impose challenges when applied to practical applications owing to the complexity of their architecture. In this study, we deeply explore RNNs due to the suitability of temporal data for building the proposed system blocks. We describe a detailed RNN of the proposed system in Section 4.3.

### 3.2. Transfer Learning and Fine-Tuning

Transfer learning intends to apply earlier acquired knowledge to accelerate the learning of new tasks [44]. In this study, let $D_{0}$ and $D_{1}$ be domains with learning tasks $T_{0}$ and $T_{1}$, respectively. The fundamental concept is to help enhance the learning of a predictive function f(·) in $T_{1}$ applying the learned knowledge extracted from $D_{0}$ and $T_{0}$, where $D_{0}$≠$D_{0}$, and/or $T_{0}$≠$T_{1}$, suggesting that domains or tasks can be different. A pre-trained model is an accumulated network earlier trained on a massive dataset. We either adopt the pre-trained model or apply transfer learning to customize this model to a given task $T_{1}$. In this paradigm, we classify the actions of humans employing transfer learning from a pre-trained network. There have been many proposed ways of customizing a pre-trained model, such as feature extraction and fine-tuning. The major variation between feature extraction and fine-tuning is that the former is done by instantiating the pre-trained model and supplementing a fully-connected classifier on top. In contrast, fine-tuning has a significant step to incrementally increase performance by repurposing the pre-trained models’ top-level layers to the new dataset. In turn, it could also possibly lead to prompt overfitting. This study employs fine-tuning to build the proposed system. We refer an interested reader to [45] for a detailed review of transfer learning.

### 3.3. On-Device Personalization

In this learning setting, we employed a fine-tuning with deep learning technique to retrain an already trained model on the cloud (that carefully trained on high-quality datasets to be as generic and unbiased as possible) to adapt to a similar mobile activity recognition problem. We only focused on two disjoint datasets that are given and the task changed, i.e., $D_{0}\cap D_{1}=⌀$ and $Y_{0}\cap Y_{1}=⌀$. The target model (on-device fine-tuned model) replicates all model designs and their parameters on the source model (on-cloud pre-trained model), except the output layer, and fine-tunes these parameters based on $D_{1}$. Contrarily, the output layer of the target model needs to be trained from scratch. In some exceptional cases, when fine-tuning is performed for $D_{1}$, it can cover a part of the original one $D_{0}$. However, to simplify notations, we ignore that parts of $D_{1}$ can already be included in $D_{0}$. Using this technique, we can create a personalized experience for the user on the device while overcoming limited training data and computational resources. For example, returning personalize estimation activities as feedback to individual devices.

## 4. Method

This section introduces the proposed system and its learning procedure. First, we introduce an overview of the methodology. Next, we describe the dataset used to train our pre-trained model. We then provide a detailed description of the network architecture and its implementation and classification performance. Finally, we discuss an optimization process for the model.

### 4.1. Overview

The objective of our work is to apply fine-tuning using RNNs to migrate the knowledge learned from the source dataset $D_{src}$ on the cloud to the target dataset $D_{tar}$ on the device for mobile activity recognition to deliver better personalized feedback to the user, as reflected in Figure 1.

Although the activities in $D_{src}$ are mostly unrelated to “walking”, models trained on this dataset can extract more general sensor features that can help identify acceleration and the rate of rotation of the device along the three sensor axes. These similar features may be equally effective for recognizing a “walking” class. Moreover, it takes less time and requires less data than training a model from scratch. We simply selected a single fully-connected layer with softmax activation as $M_{tar}$ in this experiment based on our preliminary study’s promising results [4]. However, we recommend researchers perform several experiments to see the effect of the number of layers to freeze and the number of layers to fine-tune before adopting. To build the proposed system, we implemented six steps:

1. Let $M_{src}$ be the source model pretrained on the cloud; Let $D_{src}$ be a source dataset (i.e., large-generic activity datasets); Let $M_{tar}$ be the target model trained on individual devices; Let $D_{tar}$ be the target dataset (i.e, small-personal activity datasets);
2. Build an input pipeline for $M_{src}$ using RNNs. Then, pretrain $M_{src}$ on $D_{src}$.
3. Create $M_{tar}$. This model replicates all model designs and their parameters on $M_{scr}$, except the output layer. Assume that these $M_{scr}$’s parameters hold the knowledge learned from $D_{scr}$; this knowledge will be equally applicable to $D_{tar}$. Additionally, suppose that $M_{scr}$’s output layer closely resembles the labels of $D_{scr}$ and is consequently not used in $M_{tar}$.
4. Add an output layer with a specific output size (which is equal to the number of $D_{tar}$ categories) to $M_{tar}$. Then, randomly initialize $M_{tar}$’s parameters of this layer.
5. Train the output layer of $M_{tar}$ on $D_{tar}$ from scratch. The parameters of all remaining layers are fine-tuned based on $M_{scr}$’s parameters.
6. Execute $M_{tar}$ to make predictions based on user’s input data (i.e., smartphone sensors and user-labeled data) to recognize activities and return estimated activities as feedback to the user.

### 4.2. Dataset

Large-scale datasets are prerequisites for the successful application of fine-tuning deep neural networks in a supervised learning manner. This study employed the dataset gathered from the real-world deployment on Amazon Mechanical Turk (MTurk) (https://www.mturk.com/) as $D_{src}$ to build $M_{src}$. The procedure of labeling tasks of the dataset was similar to prior work [5]. The dataset has assessed the crowdsourced data’s validity to verify that the accuracy level is sufficiently high for application to real-world data. The experiments were carried out in January and February 2020 with 120 subjects (52 female, 68 male) between the ages of 22 and 57 years old (37.64 ± 9.37). Each person performed 19 activity classes carrying an application developed for an Android smartphone in their pockets. The dataset contains the readings of two embedded sensors commonly found in smartphones: accelerometer and gyroscope, sampled at a constant frequency rate of 20 Hz. We selected 12 activity classes from the entire categories: lying down, sitting, walking, standing, handwashing, cycling, eating, using a toilet, cleaning, in a vehicle, computer work, and cooking. Given this data, it is possible to create general-model representations based on RNNs used as an initial model in the application.

### 4.3. Network Architecture and Implementation

Following our prior works [4,8], we optimally decided on the network architecture. Our preliminary findings found that RNN is incredibly well suited for sequential data because of handling arbitrary input/output lengths and the advantage of being less feature compatible when compared to other architectures such as CNN. Therefore, we employ RNNs to build the proposed system. This study explores two sequential feature models: a simple LSTM and CNN-LSTM model for performance reference.

#### 4.3.1. Simple LSTM Model

**Figure 2:**
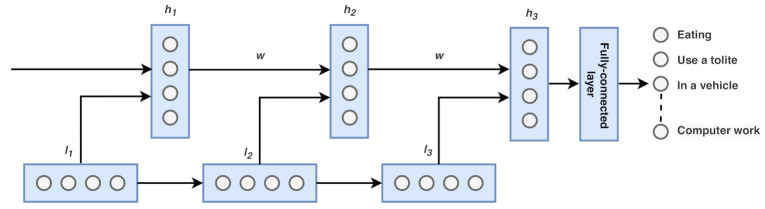
LSTM model for activity classification, where *l* is the input for each layer.

We built RNNs as the source model $M_{src}$ and prepared the sequence of vectors using a Long Short-Term Memory (LSTM) [46] layer to perform activity recognition using 3-axis acceleration sensor data available in the smartphone application as the direct input. An LSTM network is a developed RNN to solve input/output weight conflicts and avoid the vanishing gradient problem [47]. The key design of an LSTM network is to produce ways where the gradient can flow for long durations so that the time scale of combination can be modified dynamically based on the input sequence. Hence, this network has been observed remarkably successful in various activity recognition applications. Figure 2 shows our LSTM model architecture.

We created RNNs. The 3-axis acceleration and gyroscope data of each time corresponded to the dimensional input layer’s size. The number of activity classes corresponded to the dimensional output layer’s size. Each unit of each internal layer was an LSTM unit. We preprocessed the input signals since deep neural networks can learn to represent data directly from time-series data. We performed segmentation on the signals into fixed-size windows with 512 samples with a 1-second overlap. Instead of reading raw data immediately, we manually extracted valuable data from the raw sensor data. For each axis, the average and maximum and minimum values were selected as features. In sum, one representation of data had 512 time-steps × 18 features, or 9216 elements. A Rectified Linear Unit (ReLU) defined the activation function of whole layers, excluding the last fully-connected layer. A softmax function and a cross-entropy function defined the output layer’s activation function and the error function. We set $M_{src}$ holding a stacked-LSTM network that consists of two LSTM layers. This method potentially provides the hidden state at each level to perform at different timescales. They were followed by a dropout layer dedicated to reducing the model’s overfitting to the training data. The hidden layer dimension was assigned to 100. The neural network’s weight was learned using Adam [48] by setting cross-entropy as the loss function. The network was optimized by a batch size of 64 for a maximum of 15 epochs and a learning rate of 0.0001. Lastly, a fully-connected layer was adopted to describe the LSTM hidden layer’s features before a terminal output layer was employed to make predictions. The model’s output was a twelve-element vector including the probability of a given window belonging to each of the twelve activity classes.

#### 4.3.2. CNN-LSTM Model

**Figure 3:**
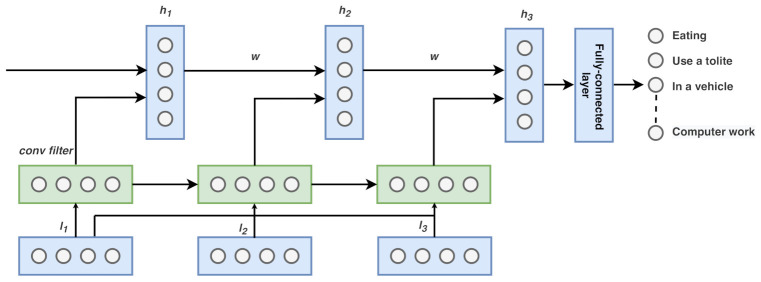
CNN-LSTM model. The input is first convolved, and fed to LSTM part.

Convolutional layers can extract valuable knowledge and discover time-series data’s internal representation, while LSTM networks efficiently recognize short-term and long-term dependencies. Our proposed CNN-LSTM model’s approach is to consolidate the benefits of these deep learning techniques efficiently to achieve a remarkably accurate classification. To this end, we designed the CNN–LSTM architecture, consisting of two main components: the CNN architecture for feature extraction and the LSTM architecture for reading the features across time steps. Figure 3 shows our CNN-LSTM model architecture.

We set the number of output, features, and window size using a similar parameter of the simple-LSTM model. We created the LSTM-CNN model that reads subsequences of the main sequence as blocks and selected features from an individual block, enabling the LSTM to understand the features extracted from each block. We divided each window of 512-time steps into four subsequences for the CNN model. As a result, the CNN model was defined to read in sequences with a length of 32-time steps and 18 features. We designed $M_{s}rc$ as having two consecutive CNN layers followed by dropout and a max-pooling layer. The whole CNN model was wrapped in a TimeDistributed (TimeDistributed layer class of Keras API; this wrapper allows us to apply a layer to every temporal slice of an input) layer to enable the same CNN model to read in each of the four subsequences in the window. The extracted features were then flattened and provided to the LSTM model to read, removing its features before a final mapping to activity was constructed. The number of filters was set to 32, and kernel size was set to 3. Similar to the simple-LSTM model, the ReLU was used as an activation function for the CNN layer. The fully connected layer beside the softmax activation function was employed to classify the activity. The network was optimized with a learning rate of 0.0001 and a batch size of 64 for a maximum of 25 epochs. The weight of the neural network was learned using Adam by setting cross-entropy as the loss function.

The simple-LSTM and CNN-LSTM model were implemented in Python using Keras v2.4.0 (https://keras.io/) with TensorFlow Core v2.0.0-rc0 (https://www.tensorflow.org/versions/r2.0/api_docs/python/tf). Then, it was converted to work with TensorFlow Lite (https://www.tensorflow.org/lite) and was ready to use in our application. Model training was run with Tesla K80 GPU in Google Colab (https://colab.research.google.com/notebooks/gpu.ipynb).

### 4.4. Classification Performance

We carried out an analysis to quantify the performance of $M_{src}$ to measure its generality before giving it to on-device. With the data prepared, we built a training and test dataset. The datasets contained different users to evaluate the robustness of the classifier to new users. We adopted the training dataset to build and validate the model and treated the test dataset as the unseen new data as if the model was in production. We used 80% for training and the remaining 20% of the data for validation. We used F-measure as a metric of accuracy.

**Figure 4:**
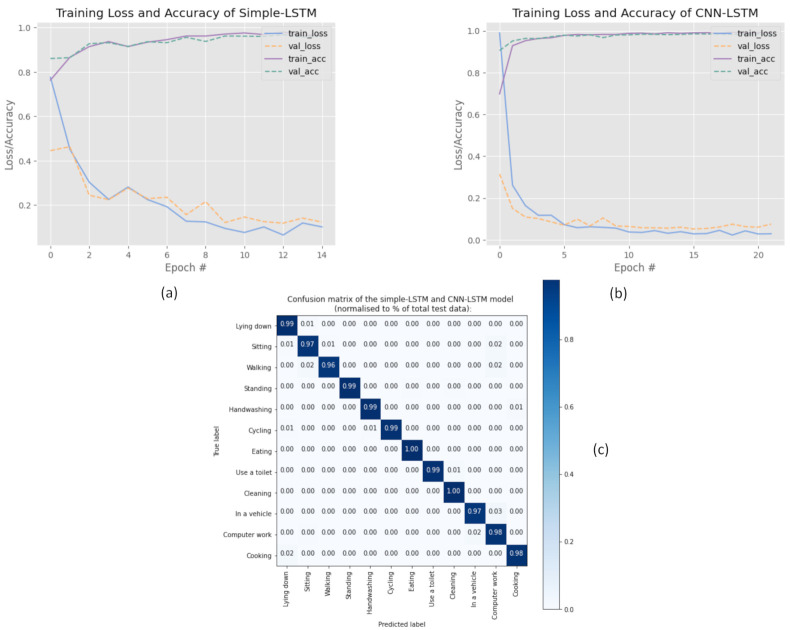
(**a**) A plot of accuracy and loss of the simple-LSTM model; (**b**) A plot of accuracy and loss of the CNN-LSTM model; (**c**) Normalized confusion matrix for the simple-LSTM and CNN-LSTM model.

Figure 4a presents the learning curves of recognition accuracy and loss by F-measure of the training and validation datasets over training epochs for the simple-LSTM model. The final epoch results show that the validation accuracy reached over 0.975 at the expense of only 0.075 validation loss. The test accuracy achieved an F-measure of 98.27%. Contrarily, Figure 4b presents the learning curves of recognition accuracy and loss by F-measure of the training and validation datasets over training epochs for the CNN-LSTM model. The final epoch results show the validation accuracy reached over 0.988 at a validation loss of only 0.046. The test accuracy achieved an F-measure of 98.78%. As a result, we can see that both models consistently perform well on the problem of accuracy, achieving an accuracy of about 98%. Overall, the results indicate that the recognition accuracy of the CNN-LSTM model was slightly higher than the simple-LSTM, with a difference of only 0.51% in F-measure for test accuracy. Additionally, Figure 4c summarizes each classifier’s performance on a set of test data using a confusion matrix with normalization by class to support the size of training for the simple-LSTM and CNN-LSTM model. Both matrices demonstrated better overall performance and could identify the movement type on a smartphone correctly. Note that we show one confusion matrix since the matrix results for the simple-LSTM are similar to that of the CNN-LSTM model.

### 4.5. Performance on a Smartphone

In real-world use, the training and inference time must be fast because our application requires immediate feedback to present to users who perform data labeling. The turned feedback should be personalized and given immediately after the task is completed. In this process, data labeling is more efficient because users’ mistakes can be corrected more quickly. Thus, we estimated the inference and training time on the smartphone. Additionally, we assume the smartphone’s resource usage such as battery damage, CPU, and memory usage is high. In that situation, it cannot be satisfactory for commercial service if its inference and training time is quick. Consequently, we examined the resources managed as inference and training performance on the smartphone.

We used Huawei P10 (Android 9.0, EMUI 9.1) for reference. The smartphone usage log was stored in the Android database. Each inference was performed at an interval of 5 min, and the total number of executions was 10 if there is no detected change in user activity. Contrarily, if there is a detected change in user activity, the inference was performed immediately. Each training was performed at an interval of 15 min, and the total number of executions was 10. Note that the standard training time depended on several factors, such as the difficulty and complexity of models, the number of samples and parameters, and the task’s design. However, typically, the model can be trained from a few seconds to a few minutes. Our analysis trained the model until the validation loss decreased well, as expected. We estimated the time for preprocessing (feature generation), training time, and inference time using a machine learning model. The average preprocessing time was 0.054 s. Table 2 presents the mean inference and training time for each model. Because the simple-LSTM model is more simple than the CNN-LSTM model, the inference and training time of the simple-LSTM model was shorter than for the CNN-LSTM model. The training time was around 54 s and 126 s for the simple-LSTM model and the CNN-LSTM model respectively. The inference time was around 0.0106 s and 0.3941 s for the simple-LSTM model and the CNN-LSTM model. Consequently, the simple-LSTM model is acceptable in real-world applications, compared to the CNN-LSTM model.

We estimated the resource usage concerning battery consumption, CPU, and memory usage of the simple-LSTM for reference. Table 3 presents the estimation results. The full battery of the Huawei P10 is 3200 milliampere-hour (mAh). The average battery consumption for each inference was 0.02300 mAh. If our application uses 10% of the total battery, the total execution number is 3200 × 0.1/0.02300 = 13,913.04. Hence, if the inference is executed every 60 s, we can use the smartphone for 13,913.04 × 60 = 834,782.4 s = 231.884 h. The average battery consumption for each training was 0.05100 mAh. If our application uses 10% of the total battery, the total execution number is 3200 × 0.1/ 0.05100 = 6274.50. Hence, if the training is performed every 60 s, we can use the smartphone for 6274.50 × 60 = 6189.3 s = 104.575 h. The average CPU usage was 5.53%, and the average memory usage was 1.03 megabytes (MB) for model inference. The average CPU usage was 22.20%, and the average memory usage was 1752.45 MB for model training. Note that we estimated the performance when only our application was performed. Consequently, a variation of the corresponding performance in real-world practice is reasonable. Still, our results indicate that resource usage is inexpensive.

In summary, the simple-LSTM model was much faster than the CNN-LSTM model, regarding the inference and training time. Moreover, the smartphone’s resource usage of the simple-LSTM model, such as battery consumption, CPU, and memory usage, is inexpensive and acceptable in real-world use. Consequently, we mainly considered the simple-LSTM model for model optimization and evaluation, as described in the following subsections.

### 4.6. Performance Optimization with Model Pruning

**Figure 5:**
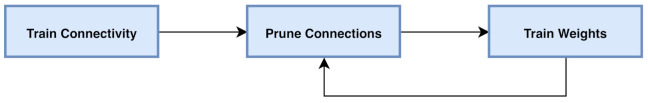
An overview of weight pruning. The compression processes the original network by pruning synapses and neurons and sharing weights back to prune connections to eliminate redundant connections to make fewer weights in its model, resulting in a minimal loss in accuracy with a 10× reduction in model size.

Deep learning model inference can be considerably computation-intensive for mobile devices, even for small input data. This section describes a model pruning technique to reduce such computation overhead, delivering the proposed system feasibly on mobile devices. Model compression is an advised approach to decrease the model size and inference computations [49]. The proposed system attempts to apply the conventional compression algorithm to minimize the complexity of optimizing on-device deep learning inference. Various optimizations have been proposed to reduce complex layers, such as pruning [10], quantization [50], and clustering [51]. We selected the magnitude-based weight pruning that performs well on mobile devices based on a collection of experiments. Figure 5 overviews the compression pipeline of a weight pruning technique. Magnitude-based weight pruning works by extracting parameters within a model that have only an insignificant impact on its predictions. Pruning gradually diminishes the number of nonzero-valued parameters in the model throughout the training process to obtain model sparsity in a deep neural network’s different connection matrices. Thereby, sparse models are sufficient at compressing, and we can ignore the zeroes during inference for latency enhancements.

This study extends the TensorFlow framework to prune the network’s connections throughout training for the simple-LSTM. We followed a gradual pruning algorithm utilized in [10] in which sparsity is grown from an initial sparsity state $s_{i}$ to a final sparsity state $s_{f}$ during *n* pruning steps, beginning at training step $t_{0}$ and with pruning frequency Δt:

(1) $$s_{t}=s_{f}+\left(s_{i}-s_{f}\right){\left(1-\frac{t-t_{0}}{n\Delta t}\right)}^{3}\;\mathrm{for}\;t\in \left\{t_{0},\;t_{0}+\Delta t,\cdots ,t_{0}+n\Delta t\right\}$$

The paired weight masks are updated each Δt steps as the network is trained to continuously enhance the network’s sparsity while allowing the network training steps to retrieve from any loss in accuracy after pruning. In our experiment, we started the model with 50% $s_{i}$ (50% zeros in weights) and end with 80% $s_{f}$. Once the model reaches the target sparsity $s_{f}$, the weight masks are no longer updated. We computed the end step to finish pruning after 15 epochs. The network was optimized with a learning rate of 0.0001 and a batch size of 64. We split 10% of the training set for the validation set. We applied pruning to the whole model and see this in the model summary. Additionally, we created a helper function to compress the models via a standard compression algorithm using gzip (gzip is a file format and a software application used for file compression and decompression) and measured the zipped size after pruning.

As a result, there was a minimal loss in test accuracy after pruning compared to the baseline. Table 4 shows the baseline test accuracy and pruned test accuracy of our simple-LSTM model. We observed that by fully pruning a model with 80% sparsity, the pruned accuracy achieved the closest performance to the baseline accuracy with a difference of approximately 0.18% in test accuracy (an accuracy of 98.27% and 98.09% for the baseline accuracy and the pruned accuracy, respectively). On the other hand, the model size was significantly decreased up to 327,212.00 bytes from pruning. The model size was 520,224.00 bytes and 193,012.00 bytes for the gzipped baseline and gzipped pruned model, respectively.

## 5. Systems Implementation

In this section, we describe the system implementation and study design to evaluate the differences between the two conditions in Table 1. The simplified input–process–output model, including data labeling, model training, and model inference for our proposed system, is summarized in Algorithm 1. The algorithm’s key component concerning the design of returning personalized feedback using on-device personalization is found in **line 27**. Note that each process is independent and can run simultaneously. In the following subsections, we detail the design rationale of each process.

**Algorithm 1:** The simplified input–process–output model for the proposed system.

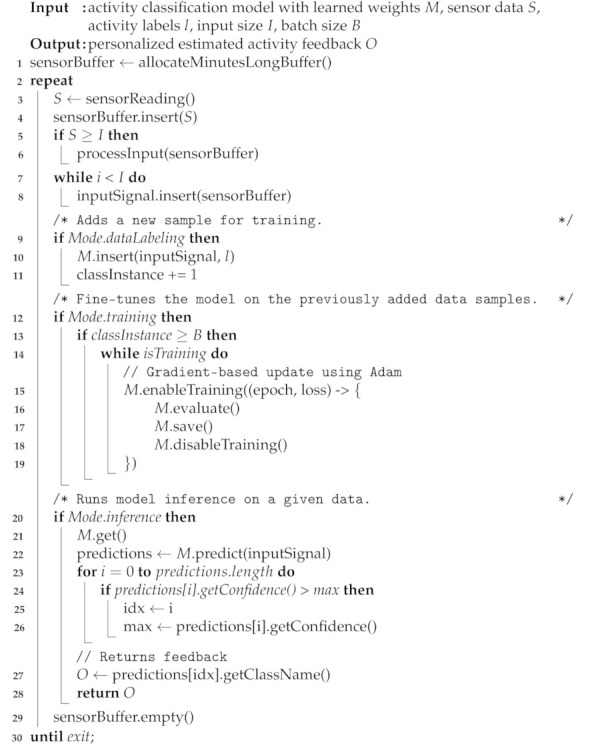



To recognize activities on the device with fine-tuning, we need to collect supervised information on sensor data activities. We implemented the FahLog (https://play.google.com/store/apps/details?id=jp.sozolab.fahlog): an Android application, written in Java with AndroidX (AndroidX is a major improvement to the original Android Support Library), which is an improvement of [25]. This application can be used for the generated models in the previous section for data collection, fine-tune training, and inference. Furthermore, we implemented a cloud server (https://fahact.sozolab.jp/), which is an improvement of [5]. It enables us to manage large-scale data from the participants and use them for evaluations. In this work, we focus on implementing the application with the required functionality for performing the proposed system. For a detailed review of the cloud-server implementation, we refer an interested reader to the abovementioned paper. Software requirement analysis of the application includes the following:

- To efficiently collect smartphone sensor data and activity labels from user’s input for activity recognition.
- To automatically fine-tune the pre-trained model with small data on individual devices.
- To deliver estimated activities gained from on-device personalization as real-time feedback through notifications.
- To support offline-first to ensure that the application functionality is unaffected by intermittent lack of a network connection.

**Figure 6:**
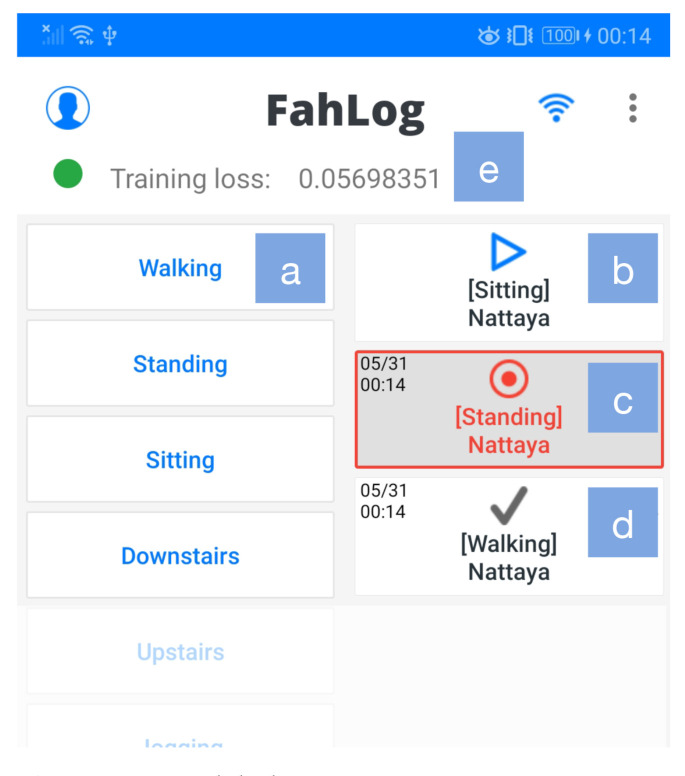
Data labeling screen.

**Figure 7:**
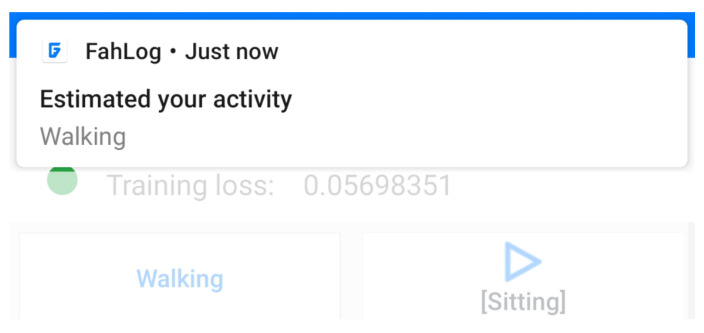
Notification displaying estimated activities.

We itemize the requirement analysis resolution and software design as follows:

- **Data labeling and smartphone sensors:** Activities are temporal data with a specific duration; it is crucial to record both the start time and the end time. For this reason, we provided the labeling screen (Figure 6), which enables a user to perform activity data labeling tasks. We detailed a written guide and associated images of the application in a user manual (https://github.com/nattafahhm/supporting-materials-sensors20/blob/master/user-manual-fahlog.pdf). The application can automatically collect smartphone sensors available on the mobile device. The sampling frequency is set at a 20-Hz, which is the standard and lowest setting. Since the participants in this study are using their smartphones, we cannot drain their battery. This configuration helped us optimize the sensing process to coordinate data generated and battery consumption, even if it had less frequent sensor readings.
- **Model fine-tuning:** Data instances keep adding their corresponding class IDs to the model cache if the data labeling is performed. Once training data is ready for use, it can be loaded into mini-batches, and the training can be initiated. In this state, data will not be immediately used for training. Instead, it will be buffered and used when the input samples’ size reaches a pre-defined batch size of the on-device model. Fine-tuning is automatically executed only every 15 min to avoid heavy computational workloads. Since the training is a simple indicator of model quality, it does not catch overfitting problems. We divided the dataset into development and test datasets and split 10% of the development set for the validation set. We then computed the loss over the validation set to ensure the model is learning what we want it to learn. Training is stopped when the validation accuracy no longer improves; the updated model overwrites the previous model. Only in this case, does it reach an accuracy percentage of over 70%. During the training process, the model is trained for a few minutes or seconds until loss decreases. The updated model is then used for inference before the next training is activated. We added functionality to show the training execution, as shown in Figure 6e. The ◯ symbol is green if training is running; otherwise, it is gray. The loss values in the panel can be observed fluctuating as the network is trained.
- **Model inference:** We reused the saved model stored in the internal device for the inference process by considering the estimates’ confidence bands. We observed the output probability of each class in a real-time manner. However, to prevent excessive interruptibility, the application stops notifying if the current activity is notified once. It resumes after 5 min or reports immediately if it detects changes in the user’s action (e.g., users in the transition from “activity a” to “activity b”). By default, all sounds and vibrations are turned on and set as a high-priority notification to ensure that the application’s notifications are notified to the user’s smartphone. Figure 7 shows an example of estimated activities on a smartphone notification.
- **Offline first:** With an offline-first approach, data are written locally on the end user’s device in the JSON format for model training and periodically uploaded to the cloud when the smartphone is connected via WiFi or mobile data for evaluations. Sensor data and activity labels are uploaded to the server by the HTTPS protocol immediately if the on-device training is successfully executed to free up space on the device due to resource constraints. Additionally, data will be deleted from the phone’s internal memory when the transmission is complete. This approach ensures that the application’s core functionality will still work in the absence of a reliable network connection.

## 6. Experiments

**Figure 8:**
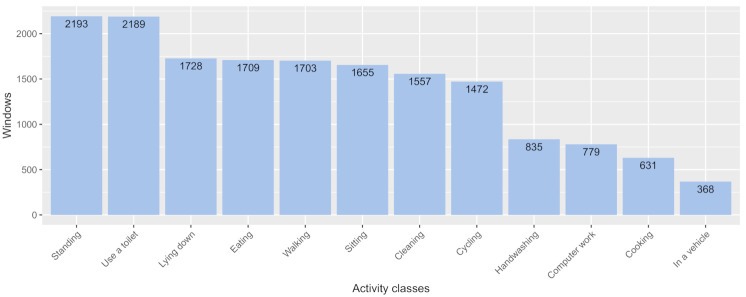
The distributions of collected data.

To verify the proper function of the protocol and data collection process and to assess the effect of the proposed method on data labeling, we performed a verification experiment. We recruited 8 volunteers who are students or alumni of a university in Thailand via social recruiting. Our post’s objective directed participants to perform an activity labeling task for four days using the provided smartphone application. Participants were required to own an Android-based smartphone with at least 5.0 or more API levels. The device was placed in a trouser’s pocket freely selected by the subject in any random orientation to simulate every phone usage. We employed a within-subject design in which all participants were exposed to every condition to help reduce errors associated with individual differences. Half of the participants were assigned to the proposed condition before they were assigned to the baseline condition. In contrast, the other half were assigned to the baseline condition before they were assigned to the proposed condition. They were asked to assign activities from the classes predefined in Figure 8 and spend 8 h per day at least (2 days per condition) on the application. The design choices and related user interface are detailed in Table 1.

Additionally, we requested participants to complete a pre-study questionnaire, focusing on demographic information and smartphone usage. We controlled for this variable by balancing participants across the two experimental conditions based on their response to minimize the learning effects across conditions. The study was conducted in early July 2020. Eight people (4 female, 4 male) between the ages of 24 and 27 years old participated in the study. A Welch’s unequal variances t-test indicated no significant difference between conditions (t = 0.65465, df = 5.069, p = 0.5412).

## 7. Activity Recognition: Evaluation and Results

This section evaluates the proposed system in depth to verify whether it can improve data labeling. We applied the simple-LSTM algorithm using the labels and sensor data collected in Section 6 for activity recognition and compared the recognition accuracy results between two conditions using the F-measure. We followed a standard activity recognition chain using a supervised learning approach—data preprocessing, segmentation, feature extraction, training, and testing. The following research questions have been defined for this study:

- **RQ1:** Can the proposed system improve data labeling in each user?
- **RQ2:** Can the proposed system improve data labeling in each activity class?

### 7.1. Data Preprocessing

We accumulated three-dimensional periodic data that incorporate acceleration and gyroscope sensors on the smartphone, recording data every 1/20 s. The axes’ norm for each row dropping in the time slot was computed to aggregate the data. Therefore, discrepancies originating from various smartphone positions/orientations at the time of the reading decreased. We later combined the periodic sensor data and activity labels without time synchronization because both are positioned on the same device. Because deep neural networks are excellent at learning representations of data directly from time-series data, we only had to perform minimal preprocessing of the input signals for the system to work properly. The data kept only the activities that correspond to each subject to avoid any unexpected or invalid activity data from affecting results. The data were then linearly interpolated to account for missing data in some of the rows. We also discarded the first and last 10 s of each activity instance for each user to account for possible transient data that were incorrectly labeled as found in practice.

Next, we transformed the raw time-series data into examples. The resulting dataset after cleanup is quite unwieldy, and it is challenging to perform a feasible analysis directly. Consequently, we segmented the data using a sliding window of 5.12 s, which has been found to be an approrpiate window of time to capture movement sequences. We then applied a 1 s displacement between consecutive windows and manually useful features from the raw sensor data to create a predictive model. For the accelerometer and gyroscope data, the average, maximum, and minimum values were extracted as features for each device’s axis. We also included the participants’ IDs for user-dependent training, as described in the next section. In total, one sample of data has (512 time-steps × 19 features), or 9728 elements. The whole dataset is composed of 16,819 activity windows, each labeled with their corresponding activity id. Figure 8 shows the distributions of collected data.

### 7.2. Evaluation Method

We developed and evaluated neural network models for multi-class classification problems. For the training algorithm, we divided the dataset into training and test sets. We used the training dataset to build and validate the model and treated the test dataset as the unseen new data. We used 20–30% of each user’s data from the beginning of the time-series and applied it for testing, and the next parts for training and validation. The training set users’ data was split into 80% for training the model and 20% for validation and hyper-parameter tuning.

Rather than applying the model to new users by comparing it with other users’ labels, we focused on the accuracy of human contributions in each condition (e.g., personal context and activities to be used by the user themself) by comparing it with the machine’s knowledge. Hence, we applied user-dependent training to show accuracy improvements for each participant in each condition without considering side effects such as different sensor positions. We utilized the F-measure as a metric of accuracy. However, the real data are highly imbalanced, as shown in Figure 8. To address this issue, we handled imbalanced classes with upsampling using the SMOTE algorithm [52] by oversampling only on the training data; none of the information in the validation data was used to create synthetic observations to make them generalizable. We then utilized the F-measure after resampling to avoid the adverse effects of class imbalances to focus on true positive samples.

The models were trained using our simple-LSTM algorithm, as described in Section 4.3. Here, we utilized the same model configuration and window size based on an earlier investigation to keep experimental evaluation unbiased due to this hyper-parameters effect. Since neural networks are stochastic, while it gives the model its adaptive ability, it is impossible to assess the model’s skill from a single evaluation. To do so, we did a slightly more detailed assessment of the model. We repeated the model’s evaluation a total of 10 times, then summarized the model’s performance across each of those runs. Additionally, we applied early-stopping during training to avoid over-fitting if the network fully converged on the training set.

### 7.3. Results

From the abovementioned research questions, we present the activity recognition accuracy results by F-measure of test data with user-independent training for two conditions from the viewpoints of (RQ1) activity recognition accuracy improvements in each user; (RQ2) activity recognition accuracy improvements in each activity class.

#### 7.3.1. RQ1: Recognition Accuracy Improvements in Each User

**Figure 9:**
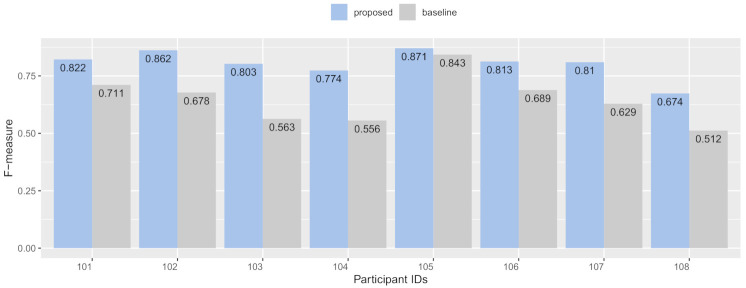
Recognition accuracy improvements in F-measure in each user.

Figure 9 shows the activity recognition accuracy by F-measure of user-dependent training for the test data. Overall, the data indicate that all participants’ recognition accuracy in the proposed condition was improved—the average recognition accuracy increased from 82% to 90% **(+16%)**. When looking at the performance of individual users, we observed the use of the proposed method increased the average recognition accuracy of F-measure by **+3%** (from 84% to 87%) to **+24%** (from 80% to 56%). All participants in the proposed condition had improved recognition accuracy, sorting by descending order as follows: The participant ID (PID) 103 had recognition accuracy improvement of **+24%** in the F-measure.

**Figure 10:**
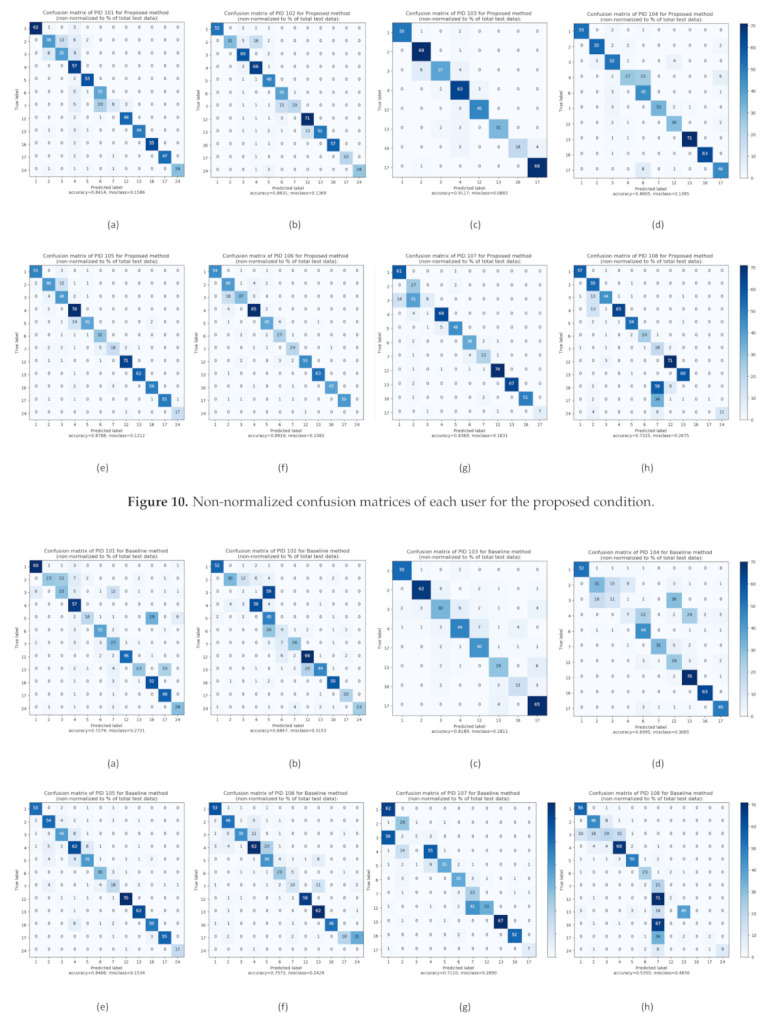
Non-normalized confusion matrices of each user for the proposed condition.

**Figure 11:**
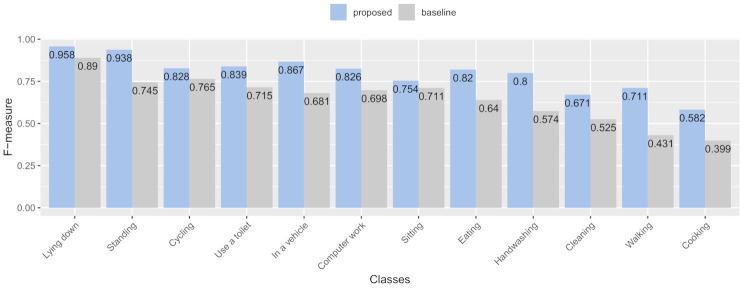
Non-normalized confusion matrices of each user for the baseline condition.

Figure 10 and Figure 11 summarizes the performance of each participant’s classifier on a set of test data using a confusion matrix with non-normalization of user-dependent training for the proposed and baseline condition, respectively. As a result, the proposed matrices were quite thick and demonstrated the overall results’ high accuracy score. In contrast, the baseline matrices were relatively sparse and explained the overall results’ low accuracy score.

#### 7.3.2. RQ2: Recognition Accuracy Improvements in Each Activity Class

**Figure 12:**
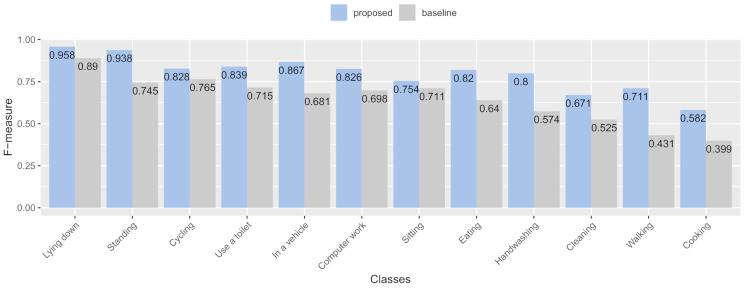
Recognition accuracy improvements in F-measure in each activity class.

Figure 12 shows the activity recognition accuracy by F-measure of each activity for the test data. Overall, the data indicate that all activities’ recognition accuracy in the proposed condition was higher than the baseline. Regarding the test data’s activity recognition accuracy with user-dependent training, we observed that the proposed condition had the highest recognition accuracy improvement of **+28%** of the F-measure in the “walking” class. The proposed condition had the next-highest recognition accuracy improvement of **+23%** in the F-measure in the “handwashing” class, followed with the improvement of **+19%** in F-measure in the “in a vehicle” and “standing” class. The remaining activities had reasonable improvement of recognition accuracy in the proposed condition as follows: the “cooking” and “eating” class had a recognition accuracy improvement of **+18%** in the F-measure; the “cleaning” class had a recognition accuracy improvement of **+15%** in the F-measure; the “computer work” class had a recognition accuracy improvement of **+13%** in the F-measure; the “use a toilet” class had a recognition accuracy improvement of **+12%** in the F-measure; the “lying down” class had a recognition accuracy improvement of **+7%** in the F-measure; the “cycling” class had a recognition accuracy improvement of **+6%** in the F-measure; the “sitting” class had a recognition accuracy improvement of **+4%** in the F-measure.

## 8. Discussion and Future Directions

In this study, we introduced a method for activity data collection utilizing on-device personalization. Although our user research is carried out on a moderate scale and for a short-term duration, the trial results have already given promising evidence that RQ1 and RQ2 were fully supported. According to the current investigation of on-device machine learning inference [24] and the official web page of TensorFlow Lite, the current utilization mainly concentrates on imaging classification, object detection, speed recognition, and natural language processing such as text classification, question answering, and smart reply. Contrarily, this research presents the application of activity recognition. We are confident that our study opens the door to an innovative application domain for on-device machine learning. Although the results are promising, there are still some weaknesses in our system. We outline remarkable limitations and discuss them below.

We assumed that the application is static or resource available for a given algorithm. However, budget resources for a specific application at runtime are not adjusted based on a predetermined estimate and can be dynamic on mobile operating systems, i.e., software platforms [53]. Thus, there is a need for research on algorithms incorporating resource-accuracy trade-off under a dynamic resource budget to choose the optimal algorithm that fits resource constraints. For instance, applying a greedy heuristic algorithm [54] to make the locally optimal choice at each stage with the intent of finding the best models or hyperparameters for multiple applications at runtime to maximize their performance jointly. This investigation can be explored in future work.

We predefined activity classes containing a fixed number of <activity, id> pairs. If the action that a user wants to input is out of the predefined list, it cannot be correctly predicted. Following prior work [15], the customizable activity class function developed is designed to be performed on the cloud and dynamically customized depending on the site server (e.g., an experimental group/facility) rather than individual users. The weak support of personalization can have a significant impact on model performance. Consequently, a customizable activity class function via the smartphone application remains to be carefully developed. However, the trade-off is the difficulty and complexity of the model design, which should be carefully considered. For example, suppose if the number of classes can change at runtime, we already need to thoroughly consider when we design the neural net’s architecture and make its classification layer large enough.

The use of transfer learning may reduce the need for massive labeled data. However, the model’s quality can be compromised if the device’s acquired data is still insufficient, such as overfitting. Several preprocessing techniques can be considered to overcome when data are sparse, such as data augmentation [55]. Data augmentation is commonly used in deep learning, where the sample size is critical for model generalization. This process stimulates new data instances that maintain the correct labels to increase the sample size when limited labeled data are available. Data augmentation usually relies on linear transformations in the spatial domain and has mainly been implemented for image recognition. However, label-preserving augmentation for time-series is much more challenging since any transformation is complicated to determine without profound domain knowledge. We are confident that the impact of data augmentation on the performance deep neuron network will introduce new challenges to be explored in future research.

Additionally, we utilized on-device fine-tuning for personalization. However, this concept can be generalized to support many other activity recognition applications. Future work should attempt to explore the impact of generalization and the tradeoffs therein. Similarly, while we employed a specific network for the two networks and achieved good training results, we may lose the optimal information if the parameter and meta parameter values are not appropriately selected. We believe that capturing several different network sizes and drawing conclusions will help achieve the greatest improvement. We intend to investigate this in future work. Further, while the accuracy level of the deployed model is sufficiently high for application to real-world data, the participants might still assign the wrong label if the model has made a few mistakes. Therefore, future research should further examine user errors that occur in such a scenario. For example, providing an accuracy percentage for participants to reduce user errors, but we need to avoid redundant information that may discourage participants. The other remaining limitations and challenges stimulate our future research; for example, we intend to attempt large-scale data collection, explore other types of optimization techniques, and further assess the usability of the proposed method with user studies.

Despite these limitations, we believe that our study is representative of a solution for the lack of accurate labels in data labeling and is an essential first step towards understanding on-device personalization in activity recognition.

## Figures and Tables

**Table 1:** Experimental design summary.

Method	Conditional detail
Proposed	Receive estimated-feedback notifications using on-device personalization.
Baseline	Receive estimated-feedback notifications using on-device inference [8].

**Table 2:** Measurement results of inference and training time.

Model	Inference Time (second)	Training Time (second)
Simple-LSTM	0.0106	54
CNN-LSTM	0.3941	126

**Table 3:** Measurement results of inference and training time.

Condition	Battery Consumption (%/times)	CPU Usage Rate (%)	Memory Usage (MB)
Inference	0.7	5.53	1.03
Training	1.6	22.20	1752.45

**Table 4:** Loss in test accuracy and a smaller model after pruning, compared to the baseline.

Condition	Test Accuracy (%)	Model Size (byte)
Baseline test accuracy	98	520,224
Pruned test accuracy	98	193,012
